# Complete Genome Sequence of *Flagellimonas* sp. Strain HMM57, Isolated from Sedimentary Layers of Crustose Coralline Algae

**DOI:** 10.1128/mra.00431-22

**Published:** 2022-07-25

**Authors:** Jang-In Shin, Man-Seok Bang, Gi-Su Lee, Ha-Na Kim, Ji Young Kim, So Hyun Park, Chung-Hun Oh

**Affiliations:** a Department of Oral Physiology, College of Dentistry, Dankook University, Cheonan, Choongnam, Republic of Korea; b Department of Medical Laser, Graduate School, Dankook University, Cheonan, Choongnam, Republic of Korea; c Research Institute for Basic Science, Jeju National University, Jeju, Republic of Korea; Montana State University

## Abstract

Here, we report the genome sequence of *Flagellimonas* sp. strain HMM57, which was isolated from sedimentary layers of crustose coralline algae in Jeju Island, South Korea. The genome is complete and consists of 4,159,450 bp, with a GC content of 38.5%, 3,616 predicted protein-coding sequences, and 70 RNA genes.

## ANNOUNCEMENT

The genus *Flagellimonas* is a member of the family *Flavobacteriaceae* in the phylum *Bacteroidetes* (1). *Flagellimonas* spp. have been reported to have been isolated from various marine algae, including *Ecklonia kurome* (2), *Ecklonia cava* (3), and *Asparagopsis taxiformisand* (4), *Hymeniacidon sinapium* (5), and surface seawater (6).

Crustose coralline algae (CCA) are rock-hard calcareous red algae that perform two major functional roles in coral reef ecosystems, that is, they contribute significantly to coral reef calcification and cementation and induce larval settlement of many benthic organisms. *Flagellimonas* sp. strain HMM57 was isolated from sedimentary layers of CCA in Hyunge-sum, Jeju Island, South Korea (33°21′037″N, 126°31′409″E). For isolation of the bacterium, 5 g wet sediment was suspended in 50 mL of filtered sterilized seawater, plated on marine agar (MA) (Difco) plates, and then cultured aerobically at 25°C for 2 weeks. After 2 weeks, microorganisms showing different colony shapes were subcultured on new MA plates. Microorganisms with different colony shapes were subcultured on new MA plates for 4 days at 25°C. The isolated microorganisms were placed in 20% glycerol and stored frozen at −80°C.

Genomic DNA was extracted from microorganisms that had been cultured in marine broth (MB) (Difco) for 4 days at 25°C by using the Wizard genomic DNA purification kit (Promega, USA), following the protocol recommended by the manufacturer. The quantity and quality of isolated DNA were determined using a NanoDrop spectrophotometer, and then 16 rRNA gene sequencing of the isolated microorganism was performed using primers 27F/907R and 785F/1492R. As a result, the best alignment (98.74% identity) to the HMM57 strain was *Muricauda eckloniae* strain DOKDO 007 (GenBank accession number NR_043626.1).

To sequence the whole genome of *Flagellimonas* sp. strain HMM57, a SMRTbell library with a 15- to 20-kb insert size (BluePippin size selection system) was constructed with the Pacific Biosciences (PacBio) DNA template preparation kit v1.0. The genome was sequenced with the RS II sequencing platform (PacBio, USA) using a single-molecule real-time (SMRT) Cell 8Pac v3 and DNA/polymerase binding kit P6 reagents by Macrogen (Seoul, Republic of Korea) (7). In total, 123,187 PacBio subreads (average subread length, 9,518 bp; subread *N*_50_, 13,636 bp) of *Flagellimonas* sp. strain HMM57 were generated.

*De novo* assembly was performed using FALCON-integrate protocol v2.1.4 (8). As default parameters, minimum subread length of 500 bp, minimum polymerase read quality of 0.8, and minimum polymerase read length of 100 bp were used. When the ends of the contig overlap, contigs are connected to form circular DNA. The result of the assembly was 1 contig, consisting of 1 closed circular chromosome of 4,159,450 bp (GC content, 38.5%; coverage, 220×). The genomes were annotated with the NCBI Prokaryotic Genome Annotation Pipeline (PGAP) v5.3 (9), which identified 3,686 genes, 3,616 coding DNA sequences, 6 rRNAs, 60 tRNAs, and 4 noncoding RNAs on the chromosome.

### Data availability.

The complete genome sequence of *Flagellimonas* sp. strain HMM57 was deposited in GenBank under the accession number CP090004.1. The associated BioProject, BioSample, Assembly, and SRA accession numbers are PRJNA789934, SAMN24175396, ASM2139017v1, and SRR17284493, respectively.
